# Green-synthesized CdTe quantum dots: dual-action nanomaterials tackling antimicrobial resistance and cancer

**DOI:** 10.1039/d6ra00549g

**Published:** 2026-04-14

**Authors:** Prutha Golakiya, Bhakti Patel, Nikita Thakkar, Gautam Priyadarshi, Rabbani Syed, Mohd Abul Kalam, Kaid Johar SR, Geethu Prakash, Dhiraj Bhatia, Raghu Solanki, Dipak Kumar Sahoo, Ashish Patel

**Affiliations:** a Department of Life Sciences, Hemchandracharya North Gujarat University Patan-384265 Gujarat India prutha.023@gmail.com bhaktipatel2233@gmail.com nikitawork1912@gmail.com priyadarshigautam411@gmail.com uni.ashish@gmail.com; b Department of Pharmaceutics, College of Pharmacy, King Saud University Riyadh 11451 Saudi Arabia rsyed@ksu.edu.sa makalam@ksu.edu.sa; c Department of Zoology, Biomedical Technology and Human Genetics, School of Sciences, Gujarat University Ahmedabad India kaidjohar@gujaratuniversity.ac.in; d Department of Biological Sciences and Engineering, Indian Institute of Technology Gandhinagar Palaj-382355 Gujarat India 23310070@iitgn.ac.in dhiraj.bhatia@iitgn.ac.in raghu.solanki@iitgn.ac.in; e Department of Veterinary Clinical Sciences, College of Veterinary Medicine, Iowa State University Ames Iowa USA dsahoo@iastate.edu

## Abstract

Antimicrobial resistance (AMR) and cancer are major health concerns that require efficient treatment strategies. An environmentally friendly extracellular biosynthesis of cadmium telluride quantum dots (CdTe QDs) was achieved using *Paenibacillus dendritiformis*, an endophytic bacterial strain. The biosynthesized CdTe QDs exhibited optical, physicochemical, and structural characteristics that were evaluated using UV-vis and photoluminescence spectroscopies, revealing a strong green fluorescence. Its monoclinic structure was revealed by XRD, and biomolecular capping was detected using FTIR spectroscopy. The zeta (*ζ*)-potential was evaluated to check their colloidal stability and negative surface charge of the particles, while FE-SEM revealed their surface morphology. Reactive oxygen species (ROS) production can be triggered by CdTe QDs, affecting essential biomolecules and bacterial membranes. The CdTe QDs also show the largest zone of inhibition of 20 mm against amoxicillin-resistant bacterial strains, *Klebsiella pneumoniae* (AMX 87) and *Enterobacter hormaechei* (AMX 03). They additionally exhibit anticancer activity against the human cervical cancer (HeLa) cell line with an IC_50_ of 60 µg mL^−1^ and the human lung cancer (A549) cell line with an IC_50_ of 65 µg mL^−1^. These results demonstrate the potential of biosynthesized CdTe QDs as an effective nanomaterial for treating AMR and cancer.

## Abstract 2

Endophytic bacteria supernatant produced CdTe QDs from precursor salts after 9 days at room temperature. The QDs were characterized (UV, PL, SEM, EDS, FTIR) and tested for antibacterial and anticancer activity.

## Graphical abstract

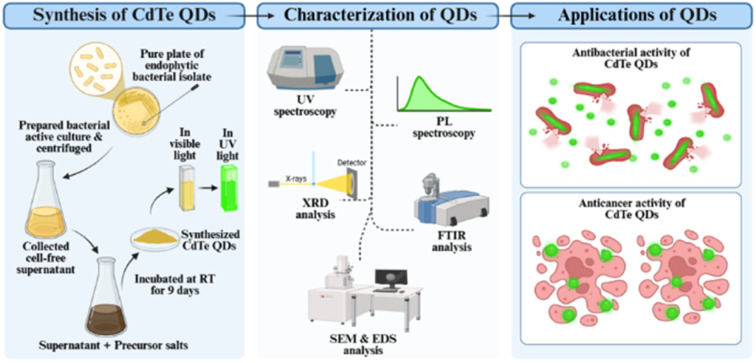

## 1. Introduction

Both antimicrobial resistance (AMR) and cancer are emerging global crises. They have attracted attention due to the limitations and failures of conventional drug therapies. Bacterial infections are becoming increasingly difficult to treat due to the growing inefficiency of conventional antibiotics. According to a systematic analysis, approximately 4.95 million mortalities were reported in 2019 worldwide due to AMR, and 1.27 million deaths occurred directly due to drug-resistant organisms causing infections. In addition to its devastating health implications, AMR creates a significant financial burden on healthcare systems worldwide. The increased duration of hospital stays, higher costs of alternative treatments, and the need for intensive care contribute to rising medical expenses. AMR may cause a 3.8% decline in global GDP by 2050, according to the World Bank estimates, which may drive millions of people towards poverty due to increased healthcare costs and low productivity.^1^

Cancer has become a serious worldwide health threat. Cancer is one of the leading causes of mortality worldwide, with around 19.3 million new cases and 10 million deaths.^2^ Lung cancer is more common in men than in women. The most dangerous type of cancer found among women is cervical cancer. Both types of cancer are the leading types of cancer worldwide, with the highest mortality rates. According to GLOBOCAN 2020, there were around 1.8 million (18.0%) deaths due to lung cancer, and its new cases were around 2.2 million (11.4%) worldwide in 2020.^3^ However, in 2020, approximately 604 127 new cases and 341 832 deaths occurred because of cervical cancer worldwide. Each year, about 13.3 new cases and 7.2 deaths from cervical cancer occur per 10 000 women worldwide.^4^ These numbers show that cervical and lung cancers are significant global health concerns.

Traditional chemotherapies often prove ineffective against various cancer types. Conventional antibiotics struggle against drug-resistant pathogens, creating an urgent need for innovative solutions to combat such infections. Quantum dots (QDs) are efficient nanomaterials in the field of nanotechnology, where materials can be synthesized from bulk to nanoscale (top-down approach) or ionic to nanoscale (bottom-up approach). QDs are zero-dimensional (0D) nanomaterials, typically spherical in shape, and possess less than 10 nm in size.^5,6^ They exhibit unique physical and chemical properties, such as quantum confinement effects. Because of these effects, they can emit fluorescence and act as an essential and alternative tool in biomedical research.^7–9^

Among the various QDs, cadmium telluride quantum dots (CdTe QDs) stand out because of their exceptional properties (minute size, high surface area-to-volume ratio, exceptional optical properties, large surface area, broad absorption spectra, strong fluorescence, strong photo-stability, non-toxicity, and biocompatibility), making them powerful tools in biomedical applications.^10,11^ They produce reactive oxygen species (ROS) under controlled conditions, which disrupt bacterial membranes and effectively eradicate multidrug-resistant bacteria.^12,13^ Additionally, CdTe QDs can be engineered to enhance antibiotic effectiveness by targeting bacterial biofilms, which are notoriously difficult to treat using conventional methods.^14^ They are easily absorbed by cells because they possess a nano size and a high surface area-to-volume ratio. Hence, they are widely utilized for the examination of cells and tissues to diagnose and cure diseases, paving the way for innovative developments in diagnostics and therapeutics.^15,16^

CdTe QDs show great promise in cancer therapy, in addition to antibacterial applications. Early detection and improved imaging resolution are made possible by their biocompatibility and targeting characteristics, which enable the accurate localization of malignant tissues. The photodynamic and photothermal properties of CdTe QDs enable the targeted elimination of cancer cells while reducing damage to the surrounding healthy tissues. These QDs provide new opportunities for non-invasive diagnostics and are tailored by utilizing their fluorescent capabilities to monitor the progression of cancer in real time.^11^ Despite these benefits, the biocompatibility and scalability of CdTe QDs are limited by the use of hazardous precursors and high temperatures in standard synthesis methods.

To overcome this problem, the present study investigates a new method for the synthesis of CdTe QDs using the supernatant of an endophytic bacterial strain. The present study first reports the use of an endophytic bacterial strain for the extracellular biosynthesis of CdTe QDs. Green synthesis offers an economical and sustainable alternative, providing controlled conditions that improve the compatibility and effectiveness of QDs in biological applications.^8,17^ Hence, the current investigation converted these results into clinical applications by evaluating the antimicrobial efficacy of CdTe QDs against antimicrobial-resistant bacterial strains, an area that is largely unexplored for such nanomaterials, thereby connecting research findings with new developments in medicine. Additionally, CdTe QDs are unexplored owing to their anticancer potential in lung cancer, so this study closes the gap between nanomaterials and medical treatments by utilizing the power of biological synthesis and nanotechnology. A revolutionary development in modern medicine, the incorporation of CdTe QDs into antibacterial and anticancer strategies opens up new possibilities for creative, effective, and long-lasting therapies.

## 2. Materials and methodology

### 2.1. Materials

Nutrient broth (n-broth) and nutrient agar (n-agar) containing peptone, sodium chloride, yeast extract, peptone B with pH (at 20 °C) 7.4 ± 0.2, sodium borohydride, and H_2_O_2_ were obtained from Hi Media (Mumbai, India). Cadmium chloride anhydrous and sodium tellurite 98% (extra pure) were obtained from Merck (Mumbai, India). Trisodium citrate and mercaptosuccinic acid (MSA) were purchased from Sigma Aldrich (Mumbai, India). (3-(4,5-Dimethylthiazol-2-yl)-2,5-diphenyltetrazolium bromide) (MTT regent), 2′,7′-dichlorofluorescein diacetate (DCFH-DA), and dimethyl sulphoxide (DMSO) were purchased from Sigma Aldrich (Saint Louis, MO, USA). Dulbecco's modified eagle medium-high glucose (DMEM-HG), 10% fetal bovine serum (FBS), phosphate buffer saline (PBS), paraformaldehyde (PFA), and Mowiol containing 4′,6-diamidino-2-phenylindole (DAPI) were procured from Thermo-Fisher Scientific (Waltham, MA, USA). The water used in the study was deionized using a Millipore system.

Human cervical cancer (HeLa) cells and human lung cancer (A549) cells were obtained from the National Centre for Cell Science (NCCS, Pune, India). Bacterial strains *Enterobacter hormaechei* sp. (AMX 03), *Klebsiella pneumoniae* sp. (AMX 72), *Shigella flexneri* sp. (AMX 22), *Klebsiella pneumoniae* (AMX 87), *Enterococcus faecalis* (AZM 07), and *Escherichia fergusonii* (AZM 37) were collected from the Gujarat Biotechnology Research Centre (GBRC) (Gujarat, India).

### 2.2. Bacterial strain isolation

The *Fagonia* sp. plant was collected from the Rann of Kutch, Gujarat (India), and an endophytic bacterial strain, *Paenibacillus dendritiformis*, was isolated from it. Plant leaves were cleaned by rinsing with Milli-Q water and then cut into fine pieces. The nutrient agar plate was calculated with 100 µL of water to test the efficacy of surface decontamination. Later, pieces of leaves were transferred onto nutrient agar plates and incubated at 37 °C. Then, by subculturing, a pure culture of organisms was obtained under the same conditions as mentioned in our previous study.^18^

### 2.3. Extracellular biosynthesis of CdTe QDs from endophytes

The endophytic bacterial strain *P. dendritiformis* was revived from master plates using sterile n-agar plates using the random streaking method and incubated at 37 °C for 24 h. Then, a pure plate was utilized to transfer a loop full of colonies into 500 mL sterile nutrient broth and incubated for 48 h at 37 °C and 150 rpm in an incubator shaker. After 48 h, the bacterial culture was centrifuged to remove bacterial cell mass for 15 min at 10 000 × *g*. The supernatant was further filtered using Whatman filter paper, and a bacterial cell-free supernatant (filtrate) was utilized to fabricate the QDs.

For extracellular biosynthesis of CdTe QDs, 44.44 mL aqueous solution of 0.04 mol per L cadmium chloride anhydrous was prepared and added into a 500 mL bacterial cell-free supernatant containing flask. Then, 1.1 g of tri-sodium citrate was directly added to the flask. An 11.11 mL aqueous solution of 0.01 mol per L sodium tellurite, 0.66 g of MSA, and 0.55 g of sodium borohydride pellets were added to the medium for the biosynthesis of CdTe QDs. After adding precursors, the mixture was mixed well, and the flask was incubated at 37 °C for 9 days under static dark conditions. Meanwhile, the OD of the solution was taken at different time intervals.^19^

Biosynthesized CdTe QDs were precipitated for 15 minutes by adding the ternary volume of organic solvent (70% ethanol) to the QD solution in a 3 : 1 ratio; then, the mixture was centrifuged for 15 min at 10 000 × *g* and 4 °C. Then, the supernatant was decanted, and the remaining pellets were washed with 70% ethanol solution, followed by deionized water. Then, the water-soluble CdTe QD solution was lyophilized, and the obtained powder was stored.

### 2.4. Characterization and analysis of biosynthesized CdTe QDs

#### 2.4.1. UV-visible spectroscopy

UV-vis analysis was carried out using a LABMAN LCD LMSP-UV1920 double beam UV-visible spectrophotometer to obtain absorption spectra at room temperature in the range of 200–800 nm. To confirm the synthesis of CdTe QDs, the sample was collected at different time intervals (0, 3rd, 6th, and 9th day).^19^

#### 2.4.2. PL (photoluminescence) spectroscopy

Photoluminescence spectroscopic analysis was performed using a photoluminescence spectrophotometer (FLS1000, Edinburgh Instruments, UK) to evaluate the emission spectra of the biosynthesized CdTe QDs.^20^

#### 2.4.3. XRD (X-ray diffraction) analysis

X-ray diffraction was utilized to analyze the average particle size and crystallinity of dried CdTe QDs using a Rigaku MiniFlex 600 instrument by bombarding CuK_α_ (*λ* = 1.540560 Å) radiation at 30 kV and 2 mA.^21^

#### 2.4.4. Fourier transform infrared (FTIR) spectroscopy analysis

To identify the functional groups and chemical bonds present on the surface of the QDs using FTIR, CdTe QDs were mixed with the KBr powder. Sample analysis of this pellet was conducted using a PerkinElmer Spectrum 65 FTIR analyzer in the range of 4000–500 cm^−1^.^21^

#### 2.4.5. Field emission scanning electron microscopy (FE-SEM) and energy dispersive X-ray spectroscopy (EDS)

The FE-SEM images of CdTe quantum dots were obtained using an Oxford EDS analyzer connected to the FE-SEM analyzer. CdTe QDs were coated with heavy metals, like gold, and then transferred onto the carbon-coated copper grid and observed across various magnifications at 20 kV for 5 µs.^22^ The elemental composition of CdTe QDs was determined using an Oxford EDS analyzer.^23^

#### 2.4.6. *ζ*-(Zeta) potential

To confirm and measure the charge of molecules present on the surface of CdTe QDs, the *ζ*-potential was measured, which was done using the Litesizer 500 zeta-potential analyzer.^24^

### 2.5. Antibacterial activity of CdTe QDs

The antibacterial activity of biosynthesized nanomaterial and conventional antibiotic (amoxicillin and azithromycin) solutions was evaluated against various bacterial strains by applying the disc diffusion technique. Activity was evaluated against different antibiotic-resistant bacterial strains, including *Enterobacter hormaechei* sp. (AMX 03), *Klebsiella pneumoniae* sp. (AMX 72), *Klebsiella pneumoniae* (AMX 87), *Shigella flexneri* sp. (AMX 22), and *Enterococcus faecalis* (AZM 07). The 1 mg mL^−1^ solutions of each CdTe QD were prepared. Active cultures of different bacteria were prepared to perform antimicrobial activity by inoculating a loop full of colonies from pure bacterial plates into nutrient broth and maintaining all of them in an incubator shaker for 24 h at 37 °C and 120 rpm. After incubation, the sterile swabs were used to evenly swab different bacterial active cultures on nutrient agar plates, and the disc loaded with 20 µL solutions of CdTe QDs and different antibiotics was placed on these plates and incubated in an upright position at 37 °C for 24 h. After incubation, the bactericidal capability of CdTe QDs and different antibiotics was tested by measuring the size of the zone of inhibition (ZOI).^25^

### 2.6. Estimation of reactive oxygen species (ROS) generation due to CdTe QDs

The bacterial cells of *Enterobacter hormaechei* sp. (AMX 03) were used to estimate ROS generation due to treatment with CdTe QDs. The bacterial culture was adjusted to the log phase, then treated with different concentrations of CdTe QDs (25, 50, and 100 µg mL^−1^) and incubated at 37 °C for 4 h. After incubation, the culture was centrifuged to collect the bacterial cells. The collected cells were washed using phosphate buffer saline (PBS), resuspended in it with DCFH-DA and incubated at 37 °C for 30 min. The excess dye was removed by washing it with PBS and labeling it as the test sample. Although untreated bacterial cells were considered the negative control, H_2_O_2_ was taken as the positive control and read using a microplate reader.

### 2.7. Anticancer activity (MTT assay) of CdTe QDs

The anticancer activity of CdTe QDs was evaluated using an MTT assay on human cervical cancer (HeLa) and human lung cancer (A549) cell lines obtained from the National Centre for Cell Science (NCCS, Pune). The cells were cultured using Dulbecco's modified Eagle medium-high glucose (DMEM-HG), which was supplemented with 10% fetal bovine serum (FBS) and 1% antibiotics. Once the cell density reached 80–90%, the experiments started.

As previously mentioned, the MTT assay was performed to assess the anti-proliferative activity of the CdTe QDs.^26^ The 96-well culture plates were inoculated with 7000 cells per well and cultured overnight. Then, the cells were treated with various concentrations (25, 50, 100, and 200 µg mL^−1^) of the CdTe QDs. The media were removed after 24 h of treatment and incubated at 37 °C for 4 h under dark conditions after the addition of 10 µL MTT reagent (5 mg mL^−1^ stock in PBS) to each well. After discarding the MTT reagent, 100 µL of DMSO was added to dissolve the formazan crystals, and the mixture was incubated for 30 minutes. Using a portable multiplate reader (Byonoy absorbance 96 plate reader), the absorbance was measured at 570 nm. Cell viability was calculated using the control group (untreated cells). CdTe QD-treated cells were used to observe morphological changes, rinsed with 1× (PBS), and then examined under an inverted microscope.^27^

### 2.8. Cellular uptake of CdTe QDs by cancer cells

CdTe QD uptake by cells was investigated in both the HeLa and A549 cell lines. For that, 50 000 cells per coverslip were seeded on the 10 mm coverslips in 24-well plates and allowed to grow overnight. The cells were visualized after 24 h for proper attachment and morphology. After two rounds of washing with 1× PBS, the cells were maintained with CdTe QDs at different concentrations of 0 µg mL^−1^, 50 µg mL^−1^, and 200 µg mL^−1^ prepared in serum-free culture media and allowed to incubate at 37 °C for 30 minutes. Following the incubation, the cells were washed 3 times with 1× PBS to remove the excess PFA and then placed on the coverslip using a Mowiol containing 4′6-diamidino-2-phenylindole (DAPI). A laser scanning confocal microscope (LSCM) was used to visualize cellular uptake at 63× magnification.

### 2.9. Statistical analysis

All experiments were conducted in triplicate (*n* = 3), and the values represented are the mean standard deviation (SD). Differences between the various groups were analyzed using one-way ANOVA, followed by a post hoc test (Tukey's HSD). SPSS (version 25.0) was used to evaluate the results, and *p* ≤ 0.05 was considered statistically significant.

## 3. Results and discussion

### 3.1. Mechanism of the biosynthesis of CdTe QDs using the bacterial supernatant

1 CdCl_2_ → Cd^2+^ + 2Cl^−^

2 Cd^2+^ + biomolecules (–COO^−^, –SH, –NH_2_) → [Cd–biomolecule]^*n*+^

3 Na_2_TeO_3_ + 2NaBH_4_ + H_2_O → NaHTe + 4B(OH)_3_ + 7H_2_↑

4 [Cd–biomolecule]^*n*+^ + Te^2−^ → CdTe (nucleus) + partially charged biomolecules

The above chemical reactions describe the biosynthesis mechanism of CdTe QDs using bacterial supernatants under controlled conditions in which bacterial biomolecules and enzymes play an important role. When cadmium chloride solution was added into the bacterial supernatant, water started dissolving them into cadmium (Cd^2+^) and chloride (Cl^−^) ions. Bacterial supernatant containing various negatively charged biomolecules (proteins, peptides, or amino acids) was attached to cadmium ions (Cd^2+^) and formed a cadmium–biomolecule complex. When sodium tellurite solution and sodium borohydride were added to the bacterial supernatant, sodium borohydride reduced sodium tellurite (Te^4+^) to telluride (Te^2−^). Then, Te^2−^ ions reacted with Cd^2+^ ions and started the formation of CdTe nuclei, while biomolecules were also partially bound on the surface of the CdTe nuclei, which provided a specific surface charge and formed a bio-capping layer. Bio-capping helps to control the toxicity of metallic CdTe QDs; due to these properties, they are widely used in biomedical applications. Similar studies show the successful synthesis of CdTe QDs from bacteria and yeast (Table 1).

**Table 1:** Comparative table for CdTe QD synthesis from various sources

No.	Source	Size	Emission	QY	Application	Reference
1	Antarctic bacteria (*Pseudomonas* spp. and *Psychrobacter* spp.)	NA	450–600 nm	NA	NA	28
2	Marine bacteria (*Bacillus pumilus* and *Serratia marcescens*)	10 nm	NA	NA	Bioimaging	29
3	Yeast cells (*Saccharomyces cerevisiae*)	2 nm	NA	NA	Anticancer activity against PC-3 (prostate carcinoma cell line)	30
4	*E. coli*	2–3 nm	450 nm	NA	NA	31
5	*E. coli*	2–3 nm	488–551 nm	15%	Bioimaging	32
6	*Paenibacillus dendritiformis*	2.3 nm	526 nm	25%	Antibacterial and anticancer	Present study

### 3.2. UV-visible spectroscopy

**Fig. 1:**
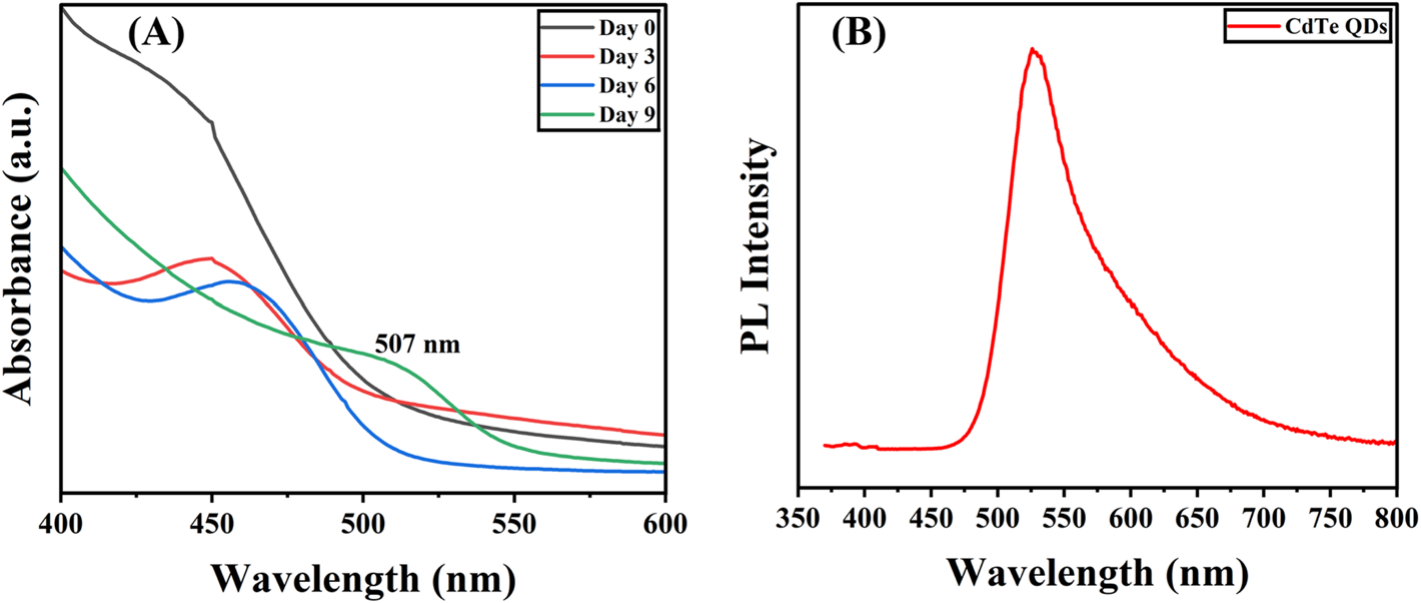
(A) UV-visible spectrum indicating the absorbance of the biosynthesized CdTe QDs at different time intervals (0, 3rd, 6th, and 9th day) over a range of wavelengths (nm), and (B) photoluminescence (PL) spectra of biosynthesized CdTe QDs showing intensity at a specific wavelength (nm).

**Fig. 2:**
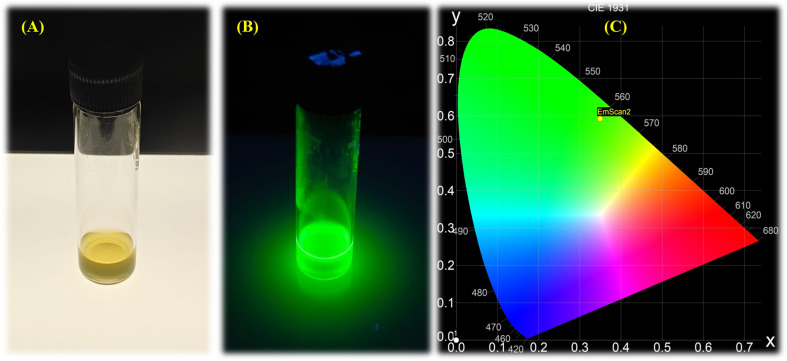
Light emission of biosynthesized CdTe QDs under (A) visible light, (B) UV light, and (C) chromaticity diagram, showing green color emission.

The biosynthesized CdTe QDs were observed for optical properties using UV-vis absorption spectroscopy over a specific time interval. Absorption peaks on day 0 and day 3 were observed at 450 nm, indicating the formation of small-sized QDs. On day 6, a slight shift in the peak was observed at a wavelength of 460 nm. The absorption peak shifted to 507 nm on day 9, which was due to an increase in the size of the QDs over the incubation period (Fig. 1A). After day 9, the solution showed no emission under visible light and started emitting bright green fluorescence when it was observed under UV light at a wavelength of 366 nm (Fig. 2A and B). For the absorption peak at 507 nm on day 9, the particle size of the biosynthesized CdTe QDs was estimated to be around 2.53 nm using the following equation:^33^

5 *D* (nm) = (9.8127 × 10^−7^)*λ*^3^ − (1.7147 × 10^−3^)*λ*^2^ + (1.0064)*λ* − (194.84),

where *D* = diameter of the particle in nm and *λ* = maximum wavelength of the absorption peak.

Vaishanav *et al.* reported a similar study, in which l-cysteine-capped CdTe QDs exhibit maximum absorption at 492 nm and a corresponding emission peak at 515 nm.^26^ Gallagher *et al.* also found that the TGA-capped CdTe QDs exhibited an absorption peak of around 510 nm, and the estimated particle size was approximately 2.3 nm.^27^ An almost similar shift in absorbance peaks over a specific incubation period was observed by Órdenes-Aenishanslins.^19^

### 3.3. Photoluminescence (PL) spectroscopy

The photoluminescence (PL) emission spectrum of the CdTe QDs showed a sharp emission peak with the highest intensity and a wavelength of around 526 nm (Fig. 1B), corresponding to the green region of the visible spectrum, and the quantum yield (QY) of the biosynthesized CdTe QDs was found to be around 25%. These results suggest the successful biosynthesis of CdTe QDs with great green fluorescent light emission properties. The CIE 1931 chromaticity graph shows the emission of green fluorescence, with an emission peak at ∼5060 nm in the green-yellow region for CdTe QDs (Fig. 2C). In the study conducted by Zhu *et al.*, they observed PL emission spectra at 540 nm for green light-emitting graphene quantum dots, and the quantum yield was found to be around 11.4%.^17^

### 3.4. XRD (X-ray diffraction) analysis

**Fig. 3:**
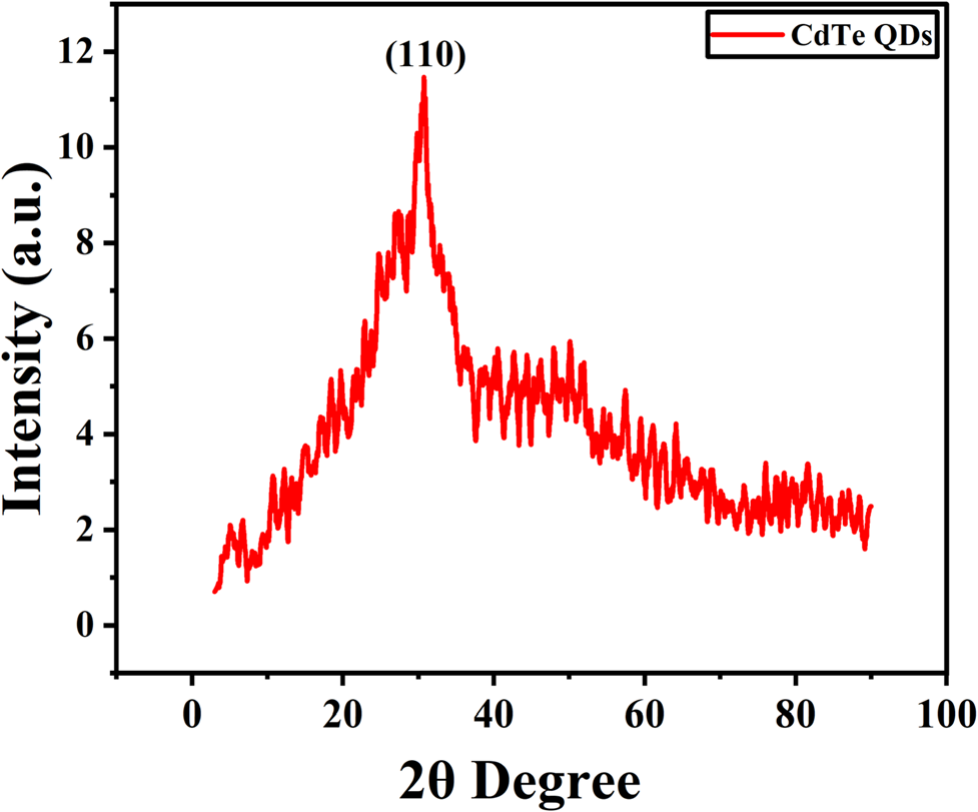
XRD diffractogram of biosynthesized CdTe QDs, where the *x*-axis represents intensity and the *y*-axis represents 2*θ* degree.

X-ray diffraction (XRD) analysis of biosynthesized CdTe QDs confirmed their nature and average particle size by showing various peaks at different angles. The sharp and broad peaks were observed at 24.8°, 30.6°, 46.2°, 52°, and 65.5° (Fig. 3). The highest peak of XRD was observed at 30.6°, which corresponds to the (110) lattice plane and confirms the monoclinic structure of biosynthesized CdTe QDs. Moreover, the average particle size of CdTe QDs was calculated using the Debye–Scherrer equation:^34^6
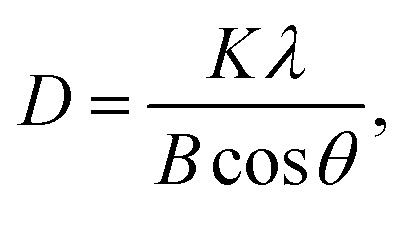
where *D* = average particle size (nm), *K* = Scherrer constant (set at 0.9 for spherical geometry), *λ* = wavelength of X-ray (for CuK_α_, 1.540560 Å), *B* = full width at half maximum (FWHM) of diffraction peak, and *θ* = Bragg's angle in radians.

The average particle size of CdTe quantum dots was obtained by applying this equation, which was 10.11 nm.

### 3.5. FTIR (Fourier transform infrared) spectroscopy analysis

**Fig. 4:**
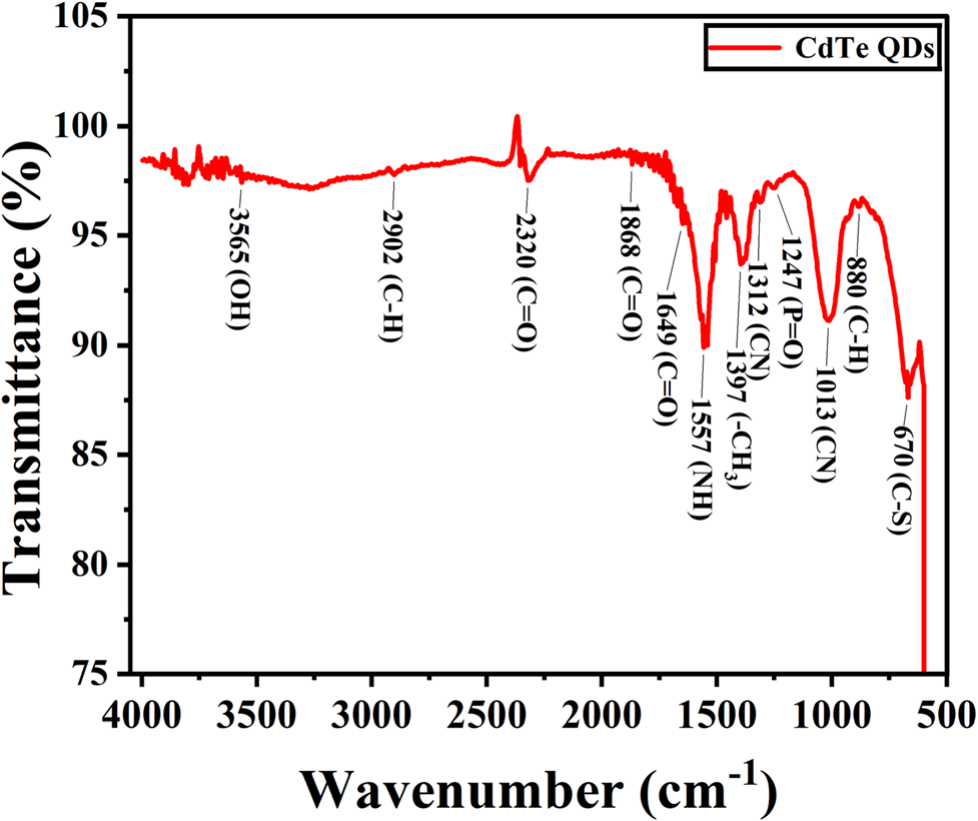
FTIR spectrum of biosynthesized CdTe QDs showing the presence of functional groups on the surface of QDs.

FTIR analysis provides an understanding of the chemical compositions and functional groups present in biosynthesized CdTe QDs by identifying the specific bonding and vibration peaks corresponding to specific groups. The FTIR spectrum of the biosynthesized CdTe QDs shows peaks at 3656 cm^−1^ (indicating alcohol or phenol bonding), 2902 cm^−1^ (alkanes containing sp^3^ bonding), 2320 cm^−1^ (atmospheric carbon dioxide gas), 1868 cm^−1^ (symmetric anhydride stretching or transition metal carbonyls), 1649 cm^−1^ (amide-I stretches), and 1557 cm^−1^ (amide-II stretches or aliphatic nitro compounds containing asymmetric stretching). Changes in amide-I and amide-II indicates modifications in the structure of proteins.^35^ The peak at 1397 cm^−1^ shows methyl stretching, 1312 cm^−1^ indicates the presence of aromatic tertiary amines, 1247 cm^−1^ corresponds to organic phosphates, 1013 cm^−1^ corresponds to primary amines, 880 cm^−1^ indicates the presence of aromatic amino acids, and 670 cm^−1^ indicates the presence of aryl thiol-ethers (Fig. 4).

Some similar FTIR peaks were observed by Fang *et al.* at around 1645 cm^−1^ (for amide-I), 1540 cm^−1^ (for amide-II), and 1232 cm^−1^ (for organic phosphates).^36^ Syed and Ahmad observed FTIR peaks at 1644 cm^−1^ (for amide-I) and 1530 cm^−1^ (for amide-II) for biosynthesized CdTe QDs from fungi *Fusarium oxysporum*,^30^ and similar peaks for amide-I and amide-II were noticed by Bao *et al.* at 1668 and 1545 cm^−1^, respectively.^16^

These peaks provide key information about specific functional groups present on the surface of synthesized CdTe QDs (Table 2), confirming the important role of bacterial enzymes, biomolecules, and MSA capping agents in the synthesis of CdTe QDs, which enhance their properties in the field of biomedicine.

**Table 2:** Prominent peaks confirm the functional groups on biosynthesized CdTe QDs

Wavenumber (cm^−1^)	Functional group symbol	Functional group name
3565	(OH)	Alcohol and phenol
2902	(CH)	Alkanes (sp^3^)
2320	(C=O)	Atmospheric carbon dioxide
1868	(C=O), M–CO	Anhydrides (symmetric stretch), transition metal carbonyls
1649	(C=O)	Amide-I
1557	(NH), NO_2_	Amide-II, aliphatic nitro compounds (asymmetric stretch)
1397	(CH_3_)	Methyl group
1312	(CN)	Aromatic tertiary amines
1247	(P=O)	Organic phosphates
1013	(CN)	Primary amines
880	(CH)	Aromatic amino acids
670	(C–S)	Aryl thiol ethers

### 3.6. FE-SEM and EDS analyses

**Fig. 5:**
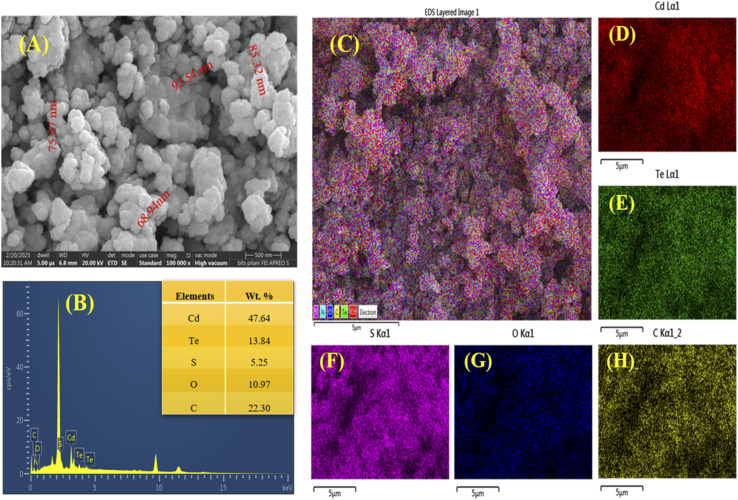
(A) SEM analysis of CdTe QDs and (B) elemental analysis of the biosynthesized CdTe QDs with (C) combined electron mapping of all elements and (D) individual electron mapping of cadmium, (E) tellurium, (F) sulfur, (G) oxygen, and (H) carbon.

The surface morphology of the biosynthesized CdTe QDs was observed using field-emission-scanning electron microscopy (FE-SEM). It can display a spherical structure and the agglomeration of CdTe QDs with a particle size ranging from 68.94 to 93.54 nm (Fig. 5). Hosnedlova *et al.* noticed the agglomerated and spherical shape of CdTe QDs in their study.^20^ However, the precise core size of QDs can be observed through TEM analysis in future studies.

The EDS spectrum displays the elemental composition of CdTe QDs; peaks for Cd, Te, S, O, and C can be observed with % weightage of 47.64, 13.84, 5.25, 10.97, and 22.30, respectively. The peaks of Cd and Te confirm the presence of CdTe QDs, and additional elements, like O or C, may appear due to surface oxidation or organic metabolites of bacteria. The peak of S may indicate the presence of a capping agent.

Almost similar results were observed in which CdTe QDs contained Cd, Te, S, and C. The peaks of C and S indicate the presence of MSA capping agent.^22^

### 3.7. Zeta (*ζ*) potential analysis

The zeta potential analysis of biologically synthesized CdTe QDs reveals a −29.5 ± 0.6 mV charge, which suggests a relatively colloidal system. A single zeta potential distribution peak is centered around −27.0 mV, indicating a uniform surface charge across the QDs. A narrower distribution indicates minimal agglomeration, and the particles appear to be well distributed. The electrophoretic mobility of CdTe QDs was −2.2975 µm cm V^−1^ s^−1^, which confirms the negative surface charge and good mobility in an electric field. The negative particle charge of the biologically synthesized CdTe QDs may be due to terminal carboxyl groups of mercaptosuccinic acid (MSA), and surface functionalization involves proteins, peptides, or biomolecules containing anionic functional groups (–COOH, –NH_2_, and –SH); these biological capping agents also impart a negative surface charge. These capping agents improve dispersion in water and provide chemically active attachment sites for the attachment of biomolecules, which suggests the potential use of CdTe QDs in biomedical applications.

These findings are comparable with previous studies on TGA-capped CdTe QDs, which showed a −30 mV zeta potential, suggesting that MSA provides similar surface stabilization as TGA-capped CdTe QDs. Najafi *et al.* found a similar work and observed that MSA-capped CdTe QDs contain a −28 mV zeta-potential value with a negative surface charge.^28^

### 3.8. Antibacterial activity

**Fig. 6:**
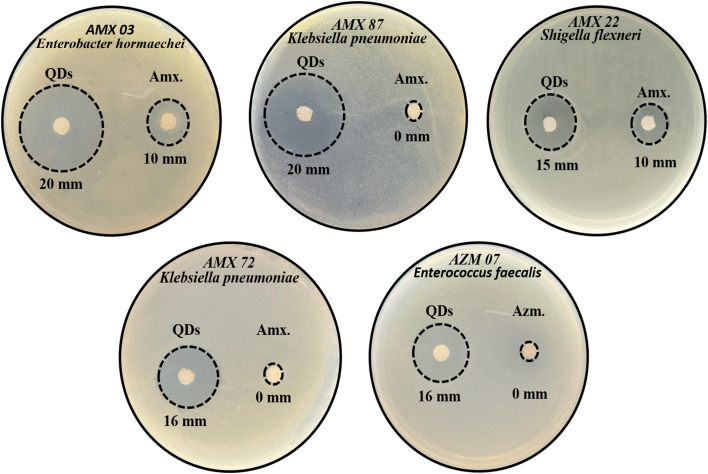
Comparative antibacterial activity of the biosynthesized CdTe QDs and standard antibiotics (amoxicillin (Amx.) and azithromycin (Azm.)) against different antimicrobial-resistant strains using the disc diffusion method, showing ZOI in mm.

The biosynthesized CdTe QDs were evaluated for their antibacterial activity against various bacterial strains, and the results were compared with the activity of the standard antibiotics [amoxicillin (Amx.) and azithromycin (Azm.)]. The results demonstrated that CdTe QDs exhibited more significant antibacterial effects than antibiotics. CdTe QDs showed the highest zone of inhibition of 20 mm for bacterial strains AMX 87 and AMX 03. Bacterial strains AMX 22 and AMX 72 produced 15 mm and 16 mm zones of inhibition (ZOI), respectively, which were larger than those observed with amoxicillin. For bacterial strain AZM 07, CdTe QDs produced a 16 mm zone of inhibition, while no zone was produced for azithromycin (Fig. 6).

Zhao *et al.* evaluated the antibacterial activity on Mueller–Hinton Agar (MHA) plates using a 0.2 mg mL^−1^ concentration of quaternized carbon quantum dots against 6 bacterial strains: *Staphylococcus aureus*, MRSA (methicillin-resistant *Staphylococcus aureus*), *Staphylococcus epidermidis*, *Enterococcus faecalis*, *Escherichia coli*, and *Pseudomonas aeruginosa*, and observed approximately 13, 12, 14, 14 and 12 mm ZOI, respectively.^31^ Safardoust-Hojaghan *et al.* evaluated ZnO/CQD nanocomposites and observed no ZOI against all tested strains, including *Staphylococcus aureus*, MRSA, *Enterococcus faecalis*, *Pseudomonas aeruginosa*, *Klebsiella pneumoniae*, vancomycin-resistant *Enterococcus faecalis* (VRE), *Shigella dysenteriae*, *Proteus vulgaris*, and *Acinetobacter pneumoniae*.^32^

### 3.9. Estimation of reactive oxygen species (ROS) generation due to CdTe QDs

**Fig. 7:**
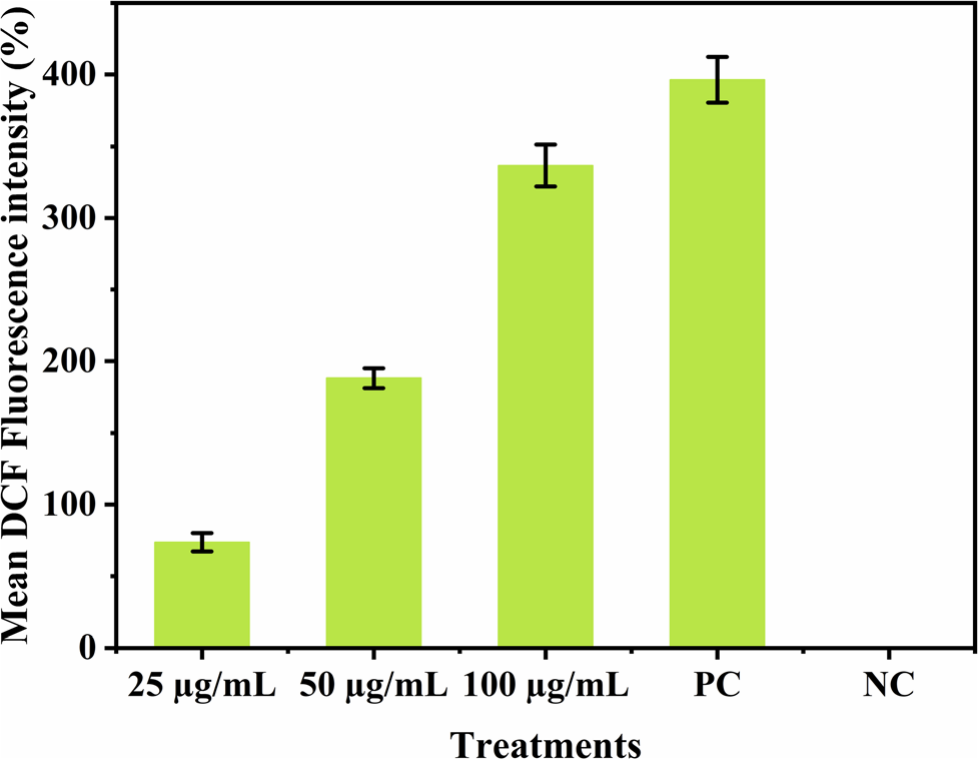
ROS generation in bacterial cells treated with various concentrations (25, 50, and 100 µg mL^−1^) of CdTe QDs, and H_2_O_2_ as a positive control (PC) and non-treated bacterial cells as a negative control (NC). Data are presented as mean DCF fluorescence in percentage, where values are represented as ±SD (*n* = 3).

The DCFH-DA assay was used to estimate ROS generation due to CdTe QD treatment. This assay presents the results in the form of the relative fluorescence intensity produced due to the reduction of DCFH-DA to DCFH and the binding of this DCFH with ROS. The results indicate the highest ROS generation in the 336.69% treatment of CdTe QDs due to the maximum fluorescence intensity. As the concentration decreases, the intensity of fluorescence decreases, indicating a decrease in ROS generation. However, the positive control gives 394.26% readings, and the negative control shows 0% fluorescence (Fig. 7). This observation suggests that the increase in CdTe QD treatment increases the ROS and simultaneously can induce oxidative stress in bacterial cells. Oxidative stress plays a crucial role in the antibacterial activity observed in the treatment of CdTe QDs.

### 3.10. Anti-proliferative activities and morphological changes in cells

**Fig. 8:**
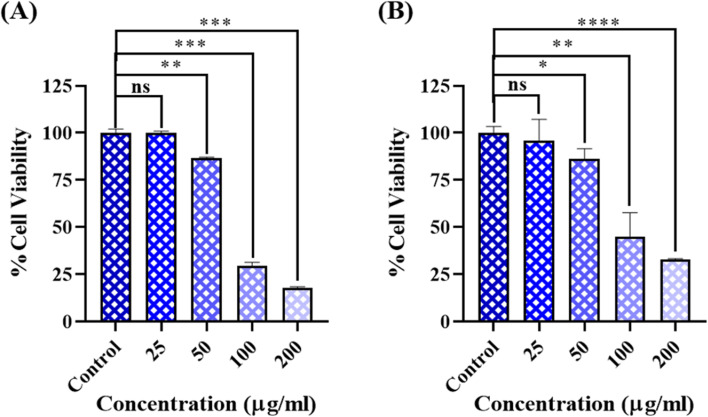
MTT assay of the biosynthesized CdTe QDs on (A) HeLa and (B) A549 cells treated with different concentrations of CdTe QDs (25, 50, 100, and 200 µg mL^−1^) for 24 h.

The anti-cancer activity of the biosynthesized CdTe QDs was assessed against HeLa cells (cervical cancer) and A549 (human lung cancer) cell lines. Due to their low toxicity, biological capping and MSA capping, solubility in water, excitation wavelength-dependent photoluminescence, and chemical stability, they stand out as potential and promising agents for cancer therapy.^37,38^ According to the results of the MTT assay, CdTe QDs showed dose-dependent cytotoxicity in both cell lines. The HeLa and A549 cells were shown to have IC_50_ values of 60 and 65 µg mL^−1^, respectively. The anticancer potential of the biosynthesized CdTe QDs was revealed by these findings, as shown in Fig. 8.

**Fig. 9:**
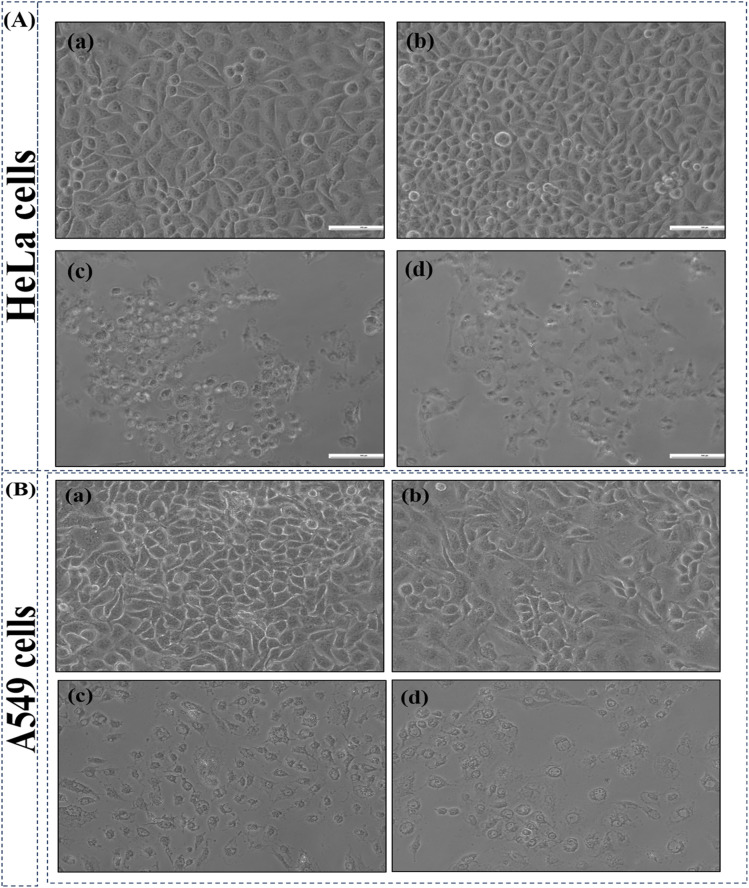
Morphological changes in HeLa and A549 cells after 24 hours of treatment with biosynthesized CdTe quantum dots (QDs). Cells were treated with QD concentrations of (a) 0 (control), (b) 25, (c) 50, (d) 100 µg mL^−1^. Images were captured using an inverted microscope.

Morphological changes in cells after the treatment of biosynthesized CdTe QDs were observed using an inverted microscope. Significant morphological and cell density changes were noted in comparison to the control group, as shown in Fig. 9. The control (untreated) cells revealed intact morphology. CdTe-treated cells displayed disrupted cell organization, cell shrinkage, round-shaped morphology, and detachment from the surface. The increase in the concentration of CdTe QDs makes the morphological changes more noticeable.

Parani *et al.* studied the anticancer activity of MSA-capped CdTe QDs with different coatings tested on HeLa cells. The IC_50_ values were 23 µg mL^−1^ for CdTe/CdS single-shell QDs (thin shell), 52 µg mL^−1^ single-shell QDs (thicker shell), and 121 µg mL^−1^ for CdTe/CdS/ZnS double-shell QDs.^39^ The A549 cells were more sensitive at the same concentrations, even with the double-shell coating. Previous studies have reported the potential application of CdTe QDs on various cell lines (Table 3).

**Table 3:** Comparative analysis of CdTe QD-mediated anticancer studies

No.	Type of synthesis	Cell line studied	IC_50_ value	Concentration	Mechanism	Reference
1	*Saccharomyces cerevisiae*-mediated CdTe QD synthesis	PC-3 (prostate carcinoma)	NA	NA	ROS generation and nuclear apoptosis	30
2	HAS-functionalized CdTe QDs	Hela (cervical cancer)	NA	0, 10, and 25 µg mL^−1^	Improved cellular uptake induces apoptosis	40
3	*Pleurotus ostreatus* mycelium-mediated CdTe QDs synthesis	MCF-7 (human breast cancer) and Colo 205 (colorectal cancer)	NA	1–5 µM	Cell membrane damage	41
4	CdTe/CdTe–CdS core shell QDs	MDA-MB468 and MCF-7 (human breast cancer)	10 µM	0, 0.1, 1, 10, 25, 50, 100, 200 µM	Apoptosis induced by ROS generation	42
5	*P. dendritiformis*-mediated extracellular CdTe QDs synthesis	HeLa (human cervical cancer) and A549 (human lung cancer) cell lines	60 µg mL^−1^ and 65 µg mL^−1^	25, 50, 100, and 200 µg mL^−1^	Apoptosis induced by ROS generation	The present study

### 3.11. Cellular uptake of QDs

**Fig. 10:**
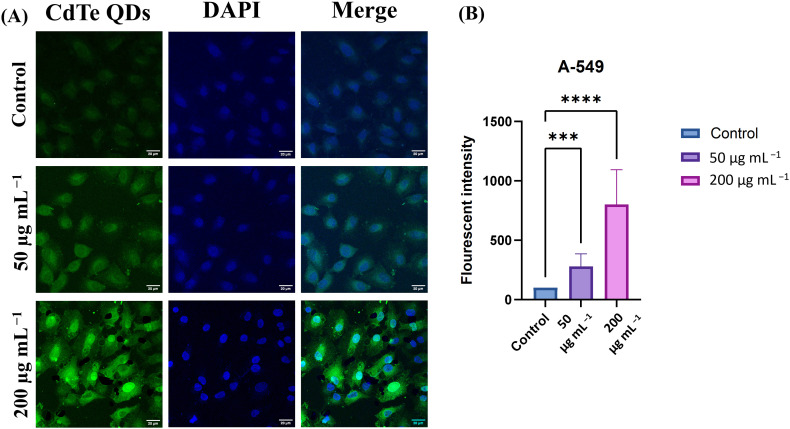
(A) Fluorescent microscopic image and (B) fluorescent intensity graph of A549 cells treated with 50 µg mL^−1^ and 200 µg mL^−1^ concentrations of CdTe QDs. Cells were observed under LSCM at 63× magnification (scale bar = 20 µm).

Due to their unique physicochemical properties, like nanoscale size, CdTe QDs can be easily taken up by cancer cells. Their high surface area, biocompatibility, and high cellular uptake make them effective drug delivery agents. CdTe QDs have been studied as potential drug delivery agents for cancer cells and bacterial infections. The cells were incubated with CdTe QDs and examined under LSCM to assess their cellular uptake. In contrast to the untreated A549 cells, the CdTe QD-treated A549 cells exhibited green fluorescence, as shown in Fig. 10. Cells treated with 200 µg mL^−1^ of CdTe QDs showed higher fluorescence intensity than those cells treated with 50 µg mL^−1^, suggesting that uptake of CdTe QDs by cells was elevated as the concentration increased. ImageJ software was utilized for the quantification of fluorescence intensity.

**Fig. 11:**
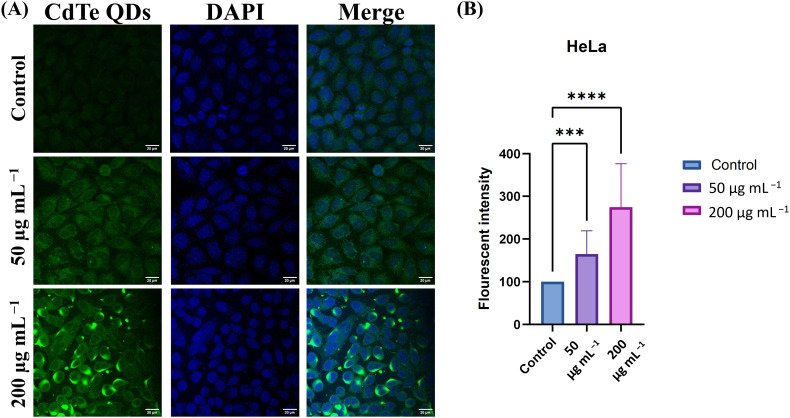
(A) Fluorescent microscopic image and (B) fluorescent intensity graph of HeLa cells treated with 50 µg mL^−1^ and 200 µg mL^−1^ concentrations of CdTe QDs and observed under LSCM at 63× magnification (scale bar = 20 µm).

HeLa cells also exhibit a similar kind of cellular uptake pattern, as shown in Fig. 11. Similar results are related to previous studies in which CdTe QDs are internalized by endocytosis in primary liver and kidney cells in mice.^43^ The anticancer effects against the HeLa and A549 cell lines may be due to the cellular uptake of CdTe QDs.

### 3.12. Mechanism of action of CdTe QDs on cytotoxicity

**Fig. 12:**
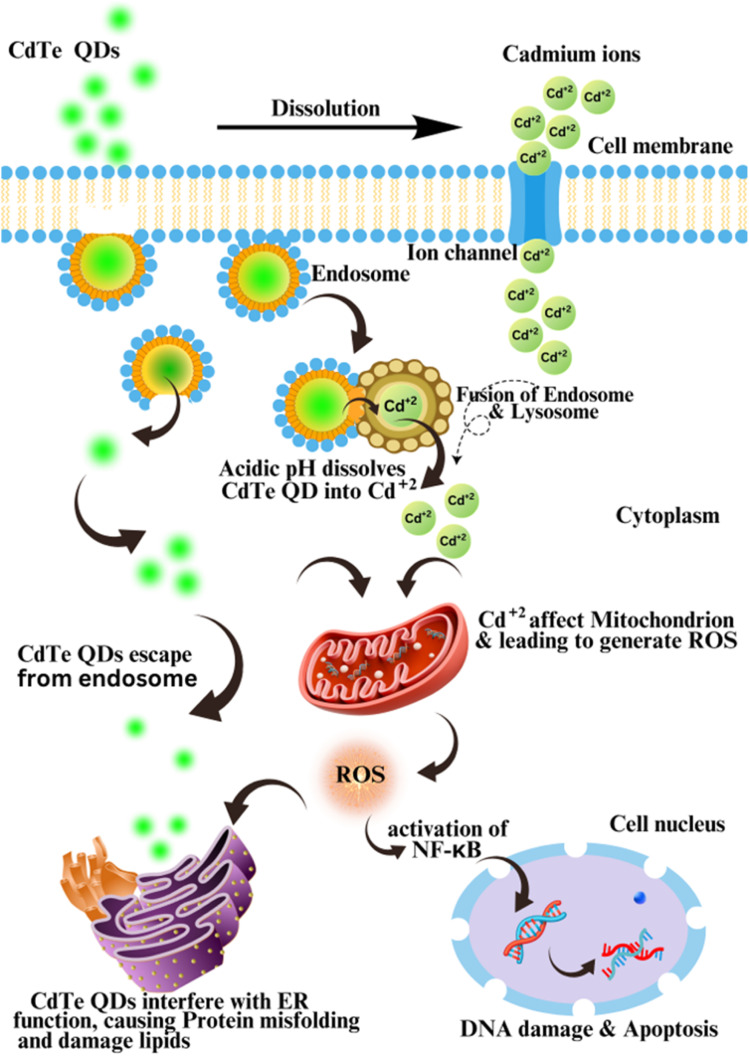
Hypothetical mechanism of induced cellular toxicity by the biosynthesized CdTe QDs.

The schematic diagram (Fig. 12) illustrates the cellular interactions and toxicological mechanisms of CdTe QDs against human cancer and AMR bacterial cells. CdTe QDs can penetrate the cell cytoplasm through a mechanism called endocytosis and are enclosed in endosomes. When endosomes are enclosed with lysosomes, because of the acidic pH of lysosomes, CdTe QDs may dissolve and convert into cadmium (Cd^2+^) ions. Extracellularly produce Cd^2+^ ions can pass through the cell membrane through the cadmium ion channel. Both the Cd^2+^ ions and CdTe QDs are probably released into the cytoplasm of the cell from the endosome and lysosome. Then, they interact with mitochondria, leading to the production of reactive oxygen species (ROS) by increasing oxidative stress. ROS include superoxide ions (O_2_^−^), hydroxyl radicals (˙OH), and hydrogen peroxide (H_2_O_2_), which disrupt the electron transport chain (ETC) in mitochondria. These ROS can destroy DNA, lipids, and proteins;^44–46^ ROS generation can also lead to the activation of the KF-κB factor, a transcription regulatory factor, which can lead to DNA disruption and ultimately apoptosis of the cell.^47^ Cd^2+^ ions, due to their positive charge, can directly interact with acidic cellular components, including the cell membrane and nuclear DNA, leading to their disruption. They lead to the misfolding of proteins and the peroxidation of lipids by interfering with the normal functions of the endoplasmic reticulum (ER). Because of their minute size and large surface area, CdTe QDs can easily penetrate through nuclear membrane pores, where they can directly interact with DNA and disrupt it.^12,48,49^

The unique properties of CdTe QDs induce cytotoxicity and generate ROS, making them potential agents for antimicrobial and anticancer applications. Their controlled toxicity, selective targeting, and biocompatibility provide new opportunities for advancements in nanomedicine.

## 4. Conclusion

This study successfully biosynthesized and characterized highly fluorescent green CdTe QDs using the supernatant of a novel endophytic bacterial strain, *Paenibacillus dendritiformis*, containing enzymes and biomolecules, under controlled environmental conditions at room temperature, highlighting their potential efficacy against antimicrobial resistance (AMR) bacterial strains and cancer cell lines. UV-visible spectroscopy confirms the synthesis of CdTe QDs from precursor salts after 9 days of incubation under static conditions by bacterial enzymes and biomolecules present in the bacterial supernatant. PL analysis confirmed the green emission spectra of the biosynthesized CdTe QDs, XRD analysis confirmed their monoclinic structure, FTIR analysis confirmed the presence of functional groups on CdTe QDs, and zeta-potential confirmed a negative particle charge uniformly present on the surface of CdTe QDs. Although morphological studies using FE-SEM revealed agglomeration, the spherical shape of CdTe QDs, and EDS analysis confirmed their elemental composition. AMR studies suggest their potential bactericidal activities against amoxicillin- and azithromycin-resistant bacterial cells. Anticancer studies against the HeLa (human cervical cancer) cell line and A549 (human lung cancer) cell line have promising potential to fight against cancer. CdTe QDs serve as an efficient alternative to conventional therapies, building a path between nanoscience and biomedicine. This study has limitations in analyzing the core size of QDs using transmission electron microscopy (TEM). Future studies should focus on the mechanistic evaluation of cell apoptosis using various methods to confirm the role of CdTe QDs in cell death. This analysis will further strengthen the understanding of the mechanism involved in the antimicrobial and anticancer activities of CdTe QDs.

## Author contributions

Prutha Golakiya: writing-original draft, methodology, investigation, formal analysis; Bhakti Patel: methodology, conceptualization, writing-review & editing, validation; Nikita Thakkar: investigation, writing-original draft, visualization, formal analysis; Gautam Priyadarshi: writing-review & editing, software, data curation, validation; Rabbani Syed: resources, validation, supervision, writing-review & editing; Mohd Abul Kalam: validation, data curation, software, visualization; Kaid Johar: validation, data curation, software, visualization; Geethu Prakash: resources, validation, supervision, writing-review & editing; Dhiraj Bhatia: writing-review & editing, software, data curation, validation; Raghu Solanki: visualization, formal analysis, writing-review & editing, investigation; Dipak Kumar Sahoo: resources, validation, supervision, writing-review & editing; and Ashish Patel: conceptualization, writing-review & editing, visualization, supervision.

## Conflicts of interest

The authors declare that they have no conflicts of interest.

## Data Availability

All data supporting the findings of this study are included within the article.
